# Augmentation therapy with minocycline in treatment-resistant depression patients with low-grade peripheral inflammation: results from a double-blind randomised clinical trial

**DOI:** 10.1038/s41386-020-00948-6

**Published:** 2021-01-28

**Authors:** Maria Antonietta Nettis, Giulia Lombardo, Caitlin Hastings, Zuzanna Zajkowska, Nicole Mariani, Naghmeh Nikkheslat, Courtney Worrell, Daniela Enache, Anna McLaughlin, Melisa Kose, Luca Sforzini, Anna Bogdanova, Anthony Cleare, Allan H. Young, Carmine M. Pariante, Valeria Mondelli

**Affiliations:** 1 King’s College London, Institute of Psychiatry Psychology and Neuroscience, Department of Psychological Medicine, London, UK; 2 National Institute for Health Research Mental Health Biomedical Research Centre, South London and Maudsley NHS Foundation Trust and King’s College London, London, UK; 3 Division of Neurogeriatrics, Department of Neurobiology, Care Sciences and Society, Karolinska Institute, Stockholm, Sweden

**Keywords:** Depression, Predictive markers, Translational research

## Abstract

This study aimed to investigate the role of baseline levels of peripheral inflammation when testing the efficacy of antidepressant augmentation with minocycline in patients with treatment-resistant depression. We conducted a 4-week, placebo-controlled, randomised clinical trial of minocycline (200 mg/day) added to antidepressant treatment in 39 patients selected for elevated levels of serum C-reactive protein (CRP ≥ 1 mg/L), *n* = 18 randomised to minocycline (M) and *n* = 21 to placebo (P). The main outcome was the change in Hamilton Depression Rating Scale (HAM-D-17) score from baseline to week 4, expressed both as mean and as full or partial response, in the overall sample and after further stratification for baseline CRP≥3 mg/L. Secondary outcomes included changes in other clinical and inflammatory measures. Changes in HAM-D-17 scores and the proportion of partial responders did not differ between study arms. After stratification for CRP levels <3 mg/L (CRP^−^) or ≥3 mg/L (CRP^+^), CRP^+^/M patients showed the largest changes in HAM-D-17 scores (mean ± SD = 12.00 ± 6.45) compared with CRP^**-**^/M (2.42 ± 3.20, *p* < 0.001), CRP^**+**^/P (3.50 ± 4.34, *p* = 0.003) and CRP^−^/P (2.11 ± 3.26, *p* = 0.006) patients, and the largest proportion (83.3%, *p* = 0.04) of partial treatment response at week 4. The threshold point for baseline CRP to distinguish responders from non-responders to minocycline was 2.8 mg/L. Responders to minocycline had higher baseline IL-6 concentrations than non-responders (*p* = 0.03); IFNγ was significantly reduced after treatment with minocycline compared with placebo (*p* = 0.03). Our data show some evidence of efficacy of add-on treatment with minocycline in MDD patients but only in those with low-grade inflammation defined as CRP ≥3 mg/L.

## Introduction

Emerging evidence of the role of the immune system in Major Depressive Disorder (MDD) has stimulated a growing interest in exploring the antidepressant properties of anti-inflammatory agents, either as monotherapy or as add-on treatment to antidepressants [1, 2]. Targeting inflammation has been proposed as a potential new strategy to treat MDD patients, in particular those who exhibit increased peripheral blood concentrations of inflammatory biomarkers and do not benefit from standard antidepressants [3].

Meta-analytical findings support a beneficial effect of anti-inflammatory treatment in depression [1], although studies so far only include Non-Steroidal Anti-Inflammatory Drugs (NSAIDs), such as COX-2 inhibitors, and cytokine inhibitors, which have direct anti-inflammatory effects, and the clinical application of these drugs in depression remains controversial for both safety and efficacy reasons. For example, NSAIDs and cytokine inhibitors increase the risk of cardiovascular adverse events [4] and the risk of infections [5], respectively, and so their safety in combination with antidepressants is still unclear. Furthermore, evidence suggests that the concurrent use of NSAIDs and antidepressants increases the risk of haemorrhage [6]. Finally, efficacy results are inconsistent, particularly for NSAIDs like COX-2 inhibitors, which, at least in some studies, showed only a modest and non-sustained antidepressant efficacy [7], or may even have an antagonistic effect on the antidepressant actions of selective serotonin reuptake inhibitors [8]. One of the reasons for such inconsistent results is that the inflammatory cascade leading to depression probably involves multiple pathways connecting the peripheral immune system to the Central Nervous System (CNS), and these may not be specifically targeted by classic anti-inflammatory treatments [1, 9].

Minocycline is a tetracycline antibiotic with broad anti-inflammatory properties and, importantly, a good penetration into the CNS through the blood-brain barrier, which accounts for its neuroprotective ability [10]. Indeed, this drug has inhibitory actions on mechanisms relevant to ‘inflammation-induced depression’, such as the kynurenine and the p-38 pathways: through the kynurenine pathway, inflammation leads to the activation of indoleamine 2,3-dioxygenase (IDO), a key enzyme in the metabolism of the serotonin precursor, tryptophan, resulting in a reduction of serotonin levels and an increase in neurotoxic metabolites [11]; and through the p-38 pathway, inflammation leads to an increase in the expression and function of the serotonin transporter, resulting in a reduction of serotonin in the synaptic space [12–14]. Moreover, evidence suggests that minocycline is also anti-oxidant and anti-apoptotic, and modulates glutamate and monoamine neurotransmission [10, 15].

Because of these unique properties of minocycline, and their relevance in the pathogenesis of depression, research has been conducted on the antidepressant efficacy of this drug, but results are not conclusive, due to the paucity and heterogeneity of studies. An initial open-label clinical trial testing the effects of adjunctive minocycline in MDD patients reported a significant improvement in depressive symptoms [16]. After that, two placebo-controlled randomised trials (RCTs) have assessed the augmentation therapy with minocycline 200 mg/day in MDD: one study found that minocycline was superior to placebo in improving Clinical Global Impression scores, quality of life and functioning, but not depressive symptoms [17], while the second, which specifically included treatment-resistant patients, found a clear effect on depressive symptoms, with a larger decrease in Hamilton Depression Rating Scale (HAM-D) scores after the administration of  minocycline compared with placebo [18]. A third RCT has tested the antidepressant properties of minocycline in HIV patients with mild-to-moderate depression, and administered as monotherapy rather than add-on treatment: the study found that minocycline was superior to placebo in improving depressive symptoms measured with the HAM-D [19]. In conclusion, as a recent meta-analysis has pointed out [20], a potential antidepressant effect has been observed for minocycline compared with placebo, but conclusions are limited by the heterogeneity of the studies. Furthermore, there is a lack of trials aiming to identify clinical subgroups that are more likely to benefit from minocycline treatment.

Of note, no study so far has considered prospectively the baseline inflammatory state of patients as a key factor moderating response to minocycline. This could be particularly relevant in view of the secondary results from an RCT with add-on treatment with Infliximab, a tumour necrosis factor (TNF)-alpha-antagonist, in patients with treatment resistant depression; in the exploratory ‘post-hoc’ stratification analyses of this study, the authors found that only patients with higher levels of C-reactive protein (CRP > 5 mg/L) showed improvement with Infliximab, while placebo was superior to Infliximab in improving depressive symptoms in those with CRP levels equal/below the identified threshold of 5 mg/L [21].

Here we present the results from our clinical trial MINDEP (MINocycline in DEPression), in which we aimed to test the role of baseline levels of peripheral inflammation in the efficacy of 4-week add-on treatment with minocycline in MDD patients not responding to antidepressant treatment. Specifically, patients were all selected for elevated levels of peripheral inflammation, measured as CRP levels ≥1 mg/L, a threshold that, as discussed in a recent meta-analysis, defines ‘elevated levels of CRP’ that are present in around 60% of depressed patients [22]. In subsequent secondary analyses, we compared the clinical outcomes of patients with CRP levels <3 mg/L or ≥3 mg/L, also based on the evidence that values above such threshold are associated with no-response to standard antidepressants [3].

We hypothesised that adjunct minocycline would be associated with greater improvement in depressive symptoms, measured at week 4 (end of treatment) when compared with placebo, and that this would be associated with normalisation of peripheral inflammatory abnormalities at week 4.

## Methods

### Overview

This was a single centre, randomised (1:1 minocycline/placebo) placebo controlled, parallel group trial of adjunctive minocycline (200 mg/day) added to ongoing treatment in patients who had failed to respond adequately to at least one antidepressant in the current depressive episode and had elevated peripheral inflammation as shown by CRP levels ≥1 mg/L. All visits took place at the Clinical Research Facility of King’s College Hospital, London.

Patients were recruited, between August 2016 and September 2019, from new referrals to primary and secondary care services linked to the South London and Maudsley NHS Foundation Trust (SLaM) and via public advertisement. All patients provided written consent after reading the information provided.

Besides antidepressants (selective serotonin reuptake inhibitors, tricyclics, monoamine oxidase inhibitors, noradrenergic and specific serotonin antagonists and serotonin noradrenaline reuptake inhibitors), current allowed medications included mood stabilisers and antipsychotics, considered on a case by case basis, as long as patients were on stable treatment for at least 6 weeks at the time they entered the study. Participants undertaking psychotherapy and other psychosocial interventions were also included.

This study was reviewed and approved by the London—Brighton & Sussex Research Ethics Committee Research Ethics Committee and by the Medicines and Healthcare products Regulatory Agency for Clinical Trial Authorisation. Trial registration: EudraCT Number 2015-003413-26. The trial ended when all participants were recruited.

### Study sample size

A previous study testing the antidepressant effect of adjunctive treatment with minocycline in 41 patients reported an improvement in HAM-D score in the minocycline group with an effect size of *d* = 1.2 (95% CI 0.39, 1.84) [18]. Assuming a similar response rate in our sample, with ~20 patients in each arm we would have more than 95% power to detect a similar reduction in HAM-D scores.

### Inclusion and exclusion criteria

Participants with MDD were selected according to the following selection criteria: (1) aged 25–60, with a current DSM-5 diagnosis of non-psychotic MDD, confirmed by the Mini International Neuropsychiatric Interview (MINI); (2) non-responders to the current antidepressant taken at therapeutic doses, as defined in the Maudsley Prescribing guidelines, for at least 6 weeks, as indicated by a current score of at least 14 on the 17- item Hamilton Depression Rating Scale (HAM-D-17); (3) tolerant to the current antidepressant and accepting augmentation with minocycline; (4) having the ability to understand and sign a written informed consent form prior to participation in any screening procedures; (5) having CRP levels ≥1 mg/L at the screening visit; and 6) having no planned changes in their current therapy for the duration of the study.

The exclusion criteria were: (1) active suicidal ideation of significant concern to require intensive monitoring by secondary psychiatry services; (2) primary diagnosis of bipolar disorder, obsessive-compulsive disorder, eating disorder, post-traumatic stress disorder, or substance/alcohol misuse disorder; (3) taking warfarin; (4) having received tetracycline within the previous 2 months, or having a history of sensitivity or intolerance to this class of drugs; (5) having an acute infection or an autoimmune or inflammatory disorder; (6) having hepatic or renal failure; and (7) taking any other psychotropic medications other than their current antidepressant that had not been approved by a study investigator prior to enrolment. All female participants did a pregnancy test before starting the study and pregnant participants and those unwilling to use an acceptable form of contraceptive throughout the study period (e.g., condoms, IUD/IUS, injection, patch, ring) were also excluded.

### Study procedure

#### Recruitment

All interested patients, either identified by clinical teams or expressing direct interest, were sent a patient information sheet which they were given time to read (at least 24 h). If they agreed to take part, they went through a pre-screening phone call to check eligibility. Then, a screening visit was set up in order to obtain signed informed consent for the study and also signed consent for the research team to have access to their medical notes.

#### Screening visit

Participants recruited to the trial underwent structured diagnostic interviews using the MINI to confirm a diagnosis of DSM-5 MDD [23]. The HAM-D-17 [24] was used to measure symptom severity and treatment response. Blood samples were also collected to test full blood count, liver and kidney function panel, and CRP levels. Vital signs, temperature, height and weight were measured as well, together with a pregnancy test for female participants.

#### Baseline visit

Within 1 month from the screening visit, eligible patients came back for the baseline visit. They were randomised to treatment with either minocycline (200 mg daily) or placebo and underwent a blood sample for measurement of biological markers and a clinical assessment including the HAM-D-17 [25], the Beck Depression Inventory II (BDI-II) [26], the Snaith–Hamilton Pleasure Scale (SHAPS) [27], the Spielberger State-Trait Anxiety Rating Scale (STAI) [28], the Clinical Global Impression (CGI) scale [29], the Brief Life Events (BLE) questionnaire [30] and the Perceived Stress Scale (PSS) [31]. Participants were also given a diary to assess their study drug compliance.

#### Randomisation

Patients were randomised (1:1 minocycline/placebo) by the method of block randomisation, stratified by gender, via a web-based randomisation system at the Clinical Trials Unit at the IoPPN. Patients and clinicians remained blind to treatment allocation. Placebo and minocycline were manufactured by Guy’s and St Thomas’ NHS Foundation Trust; minocycline was manufactured by encapsulating the Dexcel^®^-Pharma brand (Acnamino^TM^) 100 mg capsules. During the study, patients were instructed to take two capsules of the experimental medication (placebo or minocycline 100 mg) once a day. The dose was based on evidence from a previous clinical trial demonstrating a significant effect in reducing severity of depressive symptoms following treatment with minocycline with the dose of 200 mg/day in MDD [18].

#### Week 4 visit

After completion of the minocycline/placebo course, participants were assessed within 14 days of course completion. Participants underwent blood sampling for measurement of inflammatory markers, pregnancy test (female participants) and a clinical assessment with the same measures used at the baseline visit.

Day-to-day care of patients during the trial remained the responsibility of their usual consultant psychiatrist or other mental health professional. Adverse events and concomitant medications were also monitored during the entire trial.

Although data on inter-rater reliability was not formally collected, all assessments were carried out by two psychiatrists (MAN and LS) and by research assistants who are experienced Masters’ level clinical psychologists and who were trained in clinical assessments and diagnostic interviews by two authors (VM and CMP).

Overall, we screened 124 patients, out of which 49 met the inclusion criteria. From these 49, 5 patients decided not to take part in the trial; the final number of randomised patients was 44 (22:22). Five patients withdrew for different reasons (two patients experienced side effects, one was lost in follow-up and one withdrew for unknown reasons in the minocycline group; one left for family issues, in the placebo group); the final sample consisted of 39 patients, 18 in the minocycline group and 21 in the placebo group (Supplementary Fig. S1 in the supplementary material shows the Consort Flow diagram).

Table 1 shows the descriptive results for patients at the baseline, including the clinical outcome measure HAM-D-17 and the high sensitivity (hs)CRP. Patients in the two study arms were comparable for socio-demographic variables, illness duration and medication use.

**Table 1:** Socio-demographic variables.

	Minocycline *n* = 18	Placebo *n* = 21	Statistics
Age, mean (SD)	47.0 (10.0)	43.7 (10.7)	*t* = 0.98 *p* = 0.33
Gender, F (%)	55.6	57.1	*X*^2^ = 0.01 *p* = 0.92
Ethnicity, White (%)	72.2	95.3	*X*^2^ = 7.98 *p* = 0.33
BMI, mean (SD)	31.0 (6.8)	31.6 (6.2)	*t* = −0.28 *p* = 0.78
Current Smoker, yes (%)	22.2 (*n* = 4)	33.3 (*n* = 7)	*X*^2^ = 0.59 *p* = 0.44
Alcohol units per week, mean (SD)	7.2 (10.3)	9.7 (9.9)	*t* = −0.69 *p* = 0.49
Current medication^a^			
(1) SSRI (%)	61.1	47.6	*X*^2^ = 4.0 *p* = 0.26
(2) OTHER AD (%)	27.8	14.3	
(3) AD + AP (%)	5.6	14.3	
(4) >2 AD (%)	5.6	23.8	
(5) AD + benzodiazepines (%)	11.1	4.8	
Months on current medication			
(1) ≤6 months (%)	17.6	35.0	*X*^2^ = 3.74 *p* = 0.15
(2) 6–12 months (%)	0.0	10.0	
(3) ≥12 months (%)	82.4	55.0	
Depression duration from onset (years, mean (SD))	21.30 (10.92)	18.05 (12.39)	*t* = 0.89 *p* = 0.38
Baseline CTQ total score, mean (SD)	52.94 (20.22)	45.86 (11.45)	*t* = 1.36 *p* = 0.18
Baseline BLE			
(1) Stressful events, yes (%)	66.7	57.1	*X*^2^ = 0.37 *p* = 0.54
(2) Number of severe events			*X*^2^ = 0.64 *p* = 0.72
• None (%)	50.0	61.9	
• One (%)	27.8	23.8	
• 2 or more	22.2	14.3	
Baseline PSS total score, mean (SD)	26.05 (4.96)	28.62 (4.90)	*t* = −1.6 *p* = 0.11
Baseline HAM-D-17 score, mean (SD)	19.06 (3.45)	17.00 (3.26)	*t* = 1.9 *p* = 0.06
Baseline hsCRP, mean (SD)	3.13 (2.52)	4.49 (5.20)	*t* = −0.98 *p* = 0.33

^a^*AD* antidepressant, *AP* antipsychotic medication, *CTQ* Childhood Trauma Questionnaire, *BLE* Brief Life Events Scale, *PSS* Perceived Stress Scale, *HAM-D-17* Hamilton Depression Rating Scale, *hsCRP* high sensitivity C-reactive protein.

Bold means that the results are statistically significant.

### Outcome measures

The primary clinical outcome was the mean change from baseline to week 4 on the HAM-D-17, including the percentage of patients who showed treatment response, defined as 50% reduction in the baseline scores [32, 33], or partial response, defined as 25% reduction in the baseline scores [34]. Secondary outcomes included changes from baseline to week 4 in inflammatory biomarkers, Beck Depression Inventory, State and Trait Anxiety Inventory, Clinical Global Impression scale, Snaith–Hamilton Pleasure Scale and Perceived Stress Scale.

### Biomarkers

From baseline and follow-up samples, we analysed serum high sensitivity (hs)CRP using a Roche Cobas 8000 [35]. Serum pro-inflammatory and anti-inflammatory cytokines, including interferon (IFN)-γ, interleukin (IL)-1β, IL-2, IL-4, IL-6, IL-8, IL-10, IL-12p70, IL-13 and TNF-α, were measured using Meso Scale Discovery (MSD) V-PLEX sandwich immunoassays, MSD Pro-inflammatory Panel 1 (human) kit [36, 37], and plates read on an MSD QuickPlex SQ 120, as previously published [38, 39]. The inter-assay coefficient of variations was <10%. The results were analysed using MSD DISCOVERY WORKBENCH analysis software. Of note, levels of IL-1β, IL-4 and IL-12p70 were below the minimum detectable value for most of the subjects, so these cytokines were not included in the statistical analyses.

### Side effects

We calculated side effects frequency as the percentage of patients experiencing a given side effect among those randomised in each study arm. As both study arms originally counted 22 patients randomised, we used the formula (n*100)/22. This allowed us to account for patients who dropped out from the study because of side effects.

### Statistical analysis

The primary analyses included a Pearson’s Chi-square test to examine the difference in percentage of treatment response or partial response (defined as 50% or 25% reduction from baseline in the HAM-D-17 score, respectively) between the two study arms, and an independent *t*-test to test differences in changes in HAM-D-17 scores between the two study arms. Finally, we further examined differences in changes in HAM-D-17 scores between patients with hsCRP above or below the cut-off 3 mg/L at baseline [3]; for this purpose, we divided the sample by patients with hsCRP ≥ 3 mg/L (hsCRP^+^) and patients with hsCRP < 3 mg/L (hsCRP^−^), and by treatment group, generating four final groups: hsCRP^+^/M (*n* = 6), hsCRP^+^/P (*n* = 12), hsCRP^−^/M (*n* = 12) and hsCRP^−^/P (*n* = 9) (see Table 2). Then, we performed a one-way ANOVA, to investigate differences among these four groups of patients in the HAM-D-17 change.

**Table 2:** (A) HAM-D-17 and CRP descriptive statistics.

	Baseline	*n*	Week 4	*n*	Baseline vs Week 4 statistics (bootstrapped)
HAM-D-17, mean (SD)					
Minocycline	19.06 (3.45)	18	13.44 (5.17)	18	***t*** = **3.74** ***p*** = **0.008**
Placebo	17.00 (3.26)	21	14.10 (5.59)	21	***t*** = **3.43** ***p*** = **0.003**
CRP+/M	21.50 (2.59)	6	9.5 (5.32)	6	***t*** = **4.55** ***p*** = **0.02**
CRP+/P	16.08 (2.91)	12	12.58 (5.45)	12	***t*** = **2.79** ***p*** = **0.03**
CRP-/M	17.83 (3.24)	12	15.42 (3.36)	12	***t*** = **2.61** ***p*** = **0.03**
CRP-/P	18.22 (4.36)	9	16.11 (5.42)	9	*t* = 1.94 *p* = 0.11
hsCRP, mean (SD)					
Minocycline	3.13 (2.52)	18	3.30 (3.24)	17	*t* = 0.41 *p* = 0.70
Placebo	4.49 (5.20)	21	4.03 (3.53)	21	*t* = 0.52 *p* = 0.61
CRP+/M	5.68 (2.95)	6	5.13 (4.84)	6	All *p* > 0.05
CRP+/P	6.62 (6.11)	12	5.86 (3.72)	12	
CRP-/M	1.85 (0.72)	12	2.30 (1.39)	11	
CRP-/P	1.75 (0.62)	9	1.59 (0.58)	9	

(B) Proportions of responders and non-responders by groups					
	HAM-D-17 improvement <25%	*n*	HAM-D-17 improvement ≥25%	*n*	Statistics
CRP+/M	16.7%	1	83.3%	5	***X***^**2**^ = **8.27** ***p*** = **0.04**
CRP+/P	41.7%	5	58.3%	7	
CRP-/M	75.0%	9	25.0%	3	
CRP-/P	77.8%	7	22.2%	2	

HAM-D-17 Hamilton Depression Rating Scale, *hsCRP* high sensitivity C-reactive protein (analysis conducted with logarithmic CRP),  *CRP*^*+*^ baseline hsCRP levels ≥ 3 mg/L, *CRP*^*−*^  baseline hsCRP levels < 3 mg/L, *M* Minocycline, *P* Placebo.

Bold means that the results are statistically significant.

All of the aforementioned analyses were conducted in both the complete dataset and using intention-to-treat approach. Specifically, we used multiple imputation to handle missing data [40, 41], generating HAM-D-17 scores at week 4 (end of treatment) for the five withdrawn participants. The procedure involved a linear regression model (automatic method set in SPSS) and generated 12 imputations, that is, equivalent to the percentage of incomplete cases, which in our study was 11.4% [42]. The imputation model included variables used in the analysis model and associated with the imputed variable, like the Study Arm, baseline CRP (*r* = 0.341, *p* = 0.034), baseline HAM-D-17 (*r* = 0.341, *p* = 0.034) and baseline STAI-S scores (*r* = 0.45, *p* = 0.005) [42]. We compared the observed and the imputed variables by tabulating the summary statistics (Supplementary Table S2 Supplementary materials) and with both parametric and non-parametric tests [42].

Finally, we conducted a Receiver Operating Characteristic (ROC) curve analysis, with both parametric and non-parametric methods, to test the ability of baseline hsCRP levels to correctly differentiate treatment response and to identify/confirm the exact threshold point at which hsCRP would correctly identify treatment response. As CRP showed a non-normal distribution (Shapiro–Wilk test = 0.001), in the parametric method we applied the natural logarithmic transformation, which was able to normalise the CRP variable (Shapiro–Wilk test = 0.892). The baseline CRP levels (i.e., measured on the day patients were randomised) were used to identify threshold in the analysis and for all statistical purposes. It should be noted that the screening CRP, used to include patients in the study, and the baseline CRP were markedly correlated, as shown by a correlation analysis (Spearman’s rho = 0.749, *p* < 0.001).

For additional analyses, bootstrapped paired *t*-test was used to examine within-group changes, and independent *t*-test was used to examine differences in changes between the two study arms. Spearman’s correlations were used to investigate correlation between changes in blood biomarkers and changes in depressive symptoms. We performed Wilcoxon Signed-Rank and Mann–Whitney *U* tests to investigate differences within and between study arm in blood biomarkers raw values from baseline to week 4.

In terms of potential covariates, the 2 study arms did not differ in age, BMI, gender, ethnicity, tobacco and alcohol consumption. Moreover, even if, as expected, BMI was correlated with baseline CRP in the whole sample, (Spearman’s rho = 0.498, *p* = 0.001), having a BMI higher (*n* = 22) or lower (*n* = 17) than 30 (validated threshold for obesity) did not affect HAM-D-17 change in the whole sample (*t* = 0.829, *p* = 0.413).

All the statistical analyses were performed using SPSS V 26.0.

## Results

### Clinical outcome

Both the minocycline and placebo group showed significant improvement in HAM-D-17 scores (bootstrapped *t* = 3.74, *p* = 0.008; *t* = 3.43 *p* = 0.003, respectively, Table 2A) and we found no significant difference between study arms in the HAM-D-17 change (*t* = 1.57, *p* = 0.13).

We could not divide our sample in treatment responders and non-responders by using the 50% improvement cut-off for the HAM-D-17, because only three patients showed such improvement in the minocycline group, and two in the placebo group. Thus, we considered the percentage of patients who showed at least a partial response, defined as 25% reduction in the baseline scores according to the Canadian Network for mood and anxiety treatment [34]. In the overall sample, 8 out of 18 patients (44.4%) in the minocycline group showed a partial improvement, compared with 9 patients out of 21 (42.9%) in the placebo group (Pearson *χ*^2^ test *χ*^2^ = 0.01, *p* = 0.92).

**Fig. 1:**
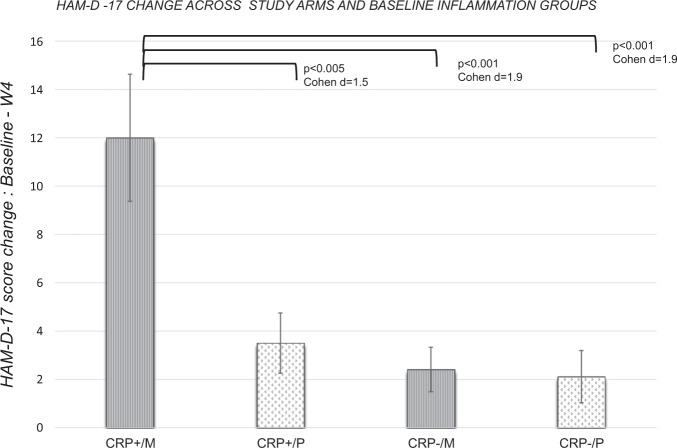
Difference in HAM-D-17 mean change, calculated as baseline scores minus week 4 scores, between patients divided by Study Arm X baseline hsCRP. Patients with hsCRP levels ≥ 3 mg/L and taking minocycline (CRP^**+**^/M) showed a significantly larger improvement compared with all other patients. HAM-D-17 = Hamilton Depression Rating Scale. CRP^+^ = baseline hsCRP levels ≥ 3 mg/L. CRP^−^ = baseline hsCRP levels <3 mg/L. M = Minocycline, P = Placebo.

When we explored differences after further stratification based on CRP levels above or below 3 mg/L, we found some evidence of efficacy for minocycline in the high inflammation group. Specifically, the one-way ANOVA showed a significant difference among the four groups of patients (CRP ≥ 3 mg/L + minocycline (CRP^+^/M) *n* = 6, CRP < 3 mg/L + minocycline (CRP^**−**^/M) *n* = 12, CRP ≥ 3 mg/L + placebo (CRP^+^/P) *n* = 12, CRP < 3 mg/L + placebo (CRP^**-**^/P) *n* = 9 (*F*_3,35_ = 8.53, *p* < 0.001). In particular, CRP^**+**^/M patients had the largest HAM-D-17 change from baseline to week 4 (mean ± SD = 12.00 ± 6.45) compared with CRP^**-**^/M (2.42 ± 3.20, *p* < 0.001, Cohen *d* = 1.9), CRP^**+**^/P (3.50 ± 4.34, *p* = 0.002, Cohen *d* = 1.5) and CRP^**-**^/P (2.11 ± 3.26, *p* < 0.001, Cohen *d* = 1.9) patients (Bonferroni corrected, see Fig. 1).

Furthermore, the hsCRP^**+**^**/**M group had the highest proportion (83.3%, five out of six) of partial responders (Table 2B) (*χ*^2^ = 8.27, *p* = 0.04).

We repeated these analyses using an intention-to-treat (ITT) approach and multiple imputation. There were no differences between the observed and imputed HAM-D-17 mean values at week 4, as confirmed by both parametric and non-parametric tests across all 12 imputations (see Supplementary Table S2, all *p* > 0.05). After adding the imputed values for the five drop-out subjects, the two study arms (with *n* = 22 subjects each) still showed no difference in all baseline demographics. Moreover, we found very similar results compared with the complete dataset. Specifically, the independent *t*-test again found no statistically significant difference in the HAM-D-17 change between the placebo and minocycline group, although in the intention-to-treat analyses actually reached trend-level significance, suggesting a greater reduction in HAM-D-17 in the minocycline than in the placebo group (pooled *t* = 1.75, *p* = 0.08). Adding the five imputed data, the four subgroups stratified by baseline hsCRP included *n* = 8 CRP^+^/M; *n* = 14 CRP^−^/M; *n* = 12 CRP^+^/P; *n* = 10 CRP^−^/P. Multiple ANOVAs comparing the four subgroups were conducted using the 12 different imputation sets, and all confirmed the significant results of the complete dataset analysis (*F* ranging 4.15–10.04, *p* values ranging *p* < 0.001–0.012), and all confirming that the CRP^+^/M group had a higher HAM-D-17 change (pooled mean SD 10.63 ± 6.54) compared with the other three groups (CRP^−^/M = 2.82 ± 3.71; CRP^−^/P = 1.9 ± 3.55; CRP^+^ *P* = 3.50 ± 4.34).

Finally, the Chi-square test confirmed that CRP^+^/M patients made up the larger proportion (pooled = 78.7%) of those with partial response, with a *χ*^2^ range = 12.42–4.85 and a *p* value range = 0.006–0.18.

The ROC analysis with non-parametric methods revealed that the threshold point for hsCRP that best distinguishes responders from non-responders in the minocycline group was 2.8 mg/L, with an area under the ROC curve = 0.792. The same threshold was found when using parametric methods and logarithmic CRP.

In the minocycline group, patients who were partially responders had higher baseline IL-6 (Mann–Whitney *U* = 16.0, *p* = 0.03) and hsCRP levels (*U* = 13.0, *p* = 0.02) compared with no-responders. No such difference was found in the placebo group.

When we analysed the other clinical measures, we found a significant improvement in BDI-II, CGI, SHAPS, STAI-T and PSS scores both in the minocycline and the placebo study arm, with no significant differences between groups (Table 3).

**Table 3:** Within and between groups analyses in other clinical scales.

	*n*	Minocycline	*n*	Placebo	Between-groups statistics
BDI-II mean (SD)					
Baseline	18	24.27 (9.75)	21	26.71 (9.20)	
Week 4	18	17.33 (20.75)	21	20.38 (17.11)	
Mean change	18	6.94 (8.46)*	21	6.33 (7.17)*	*t* = 0.24, *p* = 0.81
CGI mean (SD)					
Baseline	17	4.41 (0.50)	20	4.30 (0.92)	
Week 4	18	3.44 (1.19)	21	3.81 (0.87)	
Mean change	17	0.94 (1.14)*	20	0.45 (0.94)**	*t* = 1.43, *p* = 0.16
PSS mean (SD)					
Baseline	18	26.05 (4.96)	21	28.61 (4.90)	
Week 4	18	21.50 (8.29)	21	24.42 (6.15)	
Mean change	18	4.55 (6.08)*	21	−4.19 (5.23)*	*t* = 0.20, *p* = 0.84
SHAPS mean (SD)					
Baseline	17	7.18 (3.69)	18	5.60 (3.50)	
Week 4	18	4.61 (4.92)	19	4.20 (4.21)	
Mean change	17	3.00 (4.00)**	18	2.00 (2.66)*	*t* = 0.88, *p* = 0.38
STAI-S mean (SD)					
Baseline	17	51.18 (11.68)	21	54.09 (8.56)	
Week 4	17	47.33 (13.77)	21	48.67 (11.19)	
Mean change	17	4.05 (11.40)	21	5.43 (8.62)*	*t* = −0.42, *p* = 0.67
STAI-T mean (SD)					
Baseline	16	57.75 (8.15)	19	59.48 (6.37)	
Week 4	16	49.69 (13.14)	21	54.31 (8.97)	
Mean change	14	5.57 (9.47)**	19	5.58 (10.18)**	*t* = 0.002, *p* = 0.99

*BDI-II* Beck Depression Inventory II, *CGI* Clinical Global Impression scale, *PSS* Perceived stress scale, * SHAPS* Snaith–Hamilton Pleasure Scale,  *STAI-S* Spielberger State-Trait Anxiety Rating Scale-State,  *STAI-T* Spielberger State-Trait Anxiety Rating Scale-Trait.

*Within-group paired t-test, *p* < 0.01; **within-group paired *t*-test, *p*  < 0.05.

Bold means that the results are statistically significant.

### Biological outcomes

hsCRP and inflammatory biomarkers showed no significant changes from baseline to week 4 (Table 2A, Table 4), except for the changes in IFN-γ levels that were significantly different between groups (Mann Whitney *U* = 105.5 *p* = 0.03), with patients taking minocycline showing a decrease in IFN-γ, but not those taking placebo (Table 4). We found no significant results when the four subgroups based on baseline CRP where compared for changes in inflammatory markers.

**Table 4:** Within and between group analyses on inflammatory biomarkers.

	Minocycline baseline *n* = 18 Week 4 *n* = 17 Mean change *n* = 17	Placebo baseline *n* = 21 Week 4 *n* = 21 Mean change *n* = 21	Between-arms statistics
IL2 mean (SD) (pg/ml)			
Baseline	0.18 (0.14)	0.14 (0.12)	
Week 4	0.22 (0.15)	0.14 (0.11)	
Mean change	−0.035 (0.12)	0.00 (0.06)	*U* = 131.5 *p* = 0.17
IL6 mean (SD) (pg/ml)			
Baseline	0.87 (0.32)	0.84 (0.44)	
Week 4	1.25 (1.7)	0.76 (0.38)	
Mean change	−0.36 (1.59)	0.07 (0.33)	*U* = 173.0 *p* = 0.88
IL8 mean (SD) (pg/ml)			
Baseline	9.2 (2.64)	10.77 (3.44)	
Week 4	11.14 (4.21)	10.57 (3.62)	
Mean change	1.76 (3.38)*	−0.19 (3.24)	*U* = 131.0 *p* = 0.16
IL10 mean (SD) (pg/ml)			
Baseline	0.30 (0.25)	0.39 (0.32)	
Week 4	0.26 (0.21)	0.43 (0.48)	
Mean change	0.04 (0.33)	−0.04 (0.19)	*U* = 153.0 *p* = 0.45
IL13 mean (SD) (pg/ml)			
Baseline	0.63 (0.49)	0.63 (0.49)	
Week4	0.49 (0.46)	0.58 (0.53)	
Mean change	0.08 (0.31)	−0.12 (0.52)	*U* = 143.0 *p* = 0.31
TNFα mean (SD) (pg/ml)			
Baseline	3.29 (0.75)	3.18 (0.65)	
Week 4	3.51 (0.78)	3.30 (0.73)	
Mean change	−0.29 (0.54)	−0.12 (0.31)	*U* = 135.0 *p* = 0.21
IFNγ mean (SD) (pg/ml)			
Baseline	2.97 (2.03)	2.51 (2.15)	
Week 4	2.21 (1.61)	2.76 (1.79)	
Mean change	0.48 (0.93)	−0.24 (1.67)	***U*** = **105.5** ***p*** = **0.03**

*IL* interleukin,  *TNF* tumour necrosis factor, *IFN* interferon.

*Within group Wilcoxon Signed Ranks test, *p* < 0.05.

Bold means that the results are statistically significant.

### Side effects

There was no significant difference in the frequency of reported adverse effects between groups. The most common reported side effects were dizziness, dyspepsia, diarrhoea, headache and nausea. Supplementary Table S1 summarises all side effects reported by participants (See supplementary material).

## Discussion

In our sample of patients selected for elevated CRP (≥1 mg/L) we found no clear difference between minocycline and placebo in improving depressive symptoms at week 4 (even if the intention-to-treat analysis found trend levels of significant difference, suggesting that the minocycline group shows a greater reduction in depressive symptoms than the placebo group, possibly indicating that a significant effect could have been found in a larger sample or with a longer treatment). However, we do demonstrate that, across different analysis approaches, there is an association between baseline levels of hsCRP indicating low-grade inflammation (hsCRP levels ≥3 mg/L) and response to minocycline, such that an increased response to minocycline was found in these patients. In particular, we found that patients with baseline hsCRP levels ≥3 mg/L have an average change of 12 points in HAM-D-17 scores from baseline to week 4, with a minimum standardised effect size of 1.5 (range: 1.5–1.9) when compared with the other groups [18]. Moreover, responders to minocycline (showing at least 25% symptoms reduction) not only have higher levels of baseline hsCRP, but also of baseline IL-6. We also found that the effect of minocycline on depressive symptoms by week 4 is mirrored by a reduction in IFN-γ levels, but not in the levels of hsCRP or other cytokines.

Overall, our results corroborate the accumulating evidence that anti-inflammatory strategies, and in particular minocycline, can have an antidepressant effect only when depression is associated with increased inflammation. Our primary hypothesis that CRP = 1 mg/L could serve as inflammatory threshold to identify response to minocycline is not strongly supported by our data, while we find robust evidence in favour of using CRP = ~3 mg/L. This has been considered the cut-off for “low-grade inflammation” which characterises over a quarter of patients with depression and can predict not only treatment-resistance to antidepressants, but also comorbid, immune related physical illnesses [22]. CRP levels ≥3 mg/L have also been associated with reduced connectivity within reward related circuits (measured with fMRI) and with alterations of glutamate metabolism [43]. This is particularly relevant considering minocycline modulation of the glutamatergic neurotransmission [10].

Our findings that levels of CRP and IL-6 are predictive of minocycline response in depression are consistent with existing evidence. For example, high baseline CRP before treatment has previously been associated with better response in MDD patients to the cytokine inhibitor Infliximab [21]. Similar to our findings, high basal levels of IL-6 predicted antidepressant efficacy of anti-inflammatory agents, including celecoxib [44] and minocycline itself, as showed in a 6-week trial in bipolar depression [45].

In contrast with both these studies, we did not find a reduction in IL-6 following minocycline administration. In particular, in the study by Savitz and colleagues, participants with bipolar depression who responded to minocycline had significantly greater decreases of IL-6 over 6 weeks of treatment when compared with non-responders. By contrast, we found no reduction in inflammatory biomarkers following minocycline administration in our sample of patients. Only changes in IFN-γ levels were significantly different in the two study arms, indicating a modest reduction in IFN-γ levels in the minocycline group compared with placebo. However, such change did not correlate with changes in any clinical measure. The reason for such discrepancy between our findings and those by Savitz and colleagues might be the shorter exposure to minocycline in our study (4 weeks vs 6 weeks) or the characteristics of the clinical sample, which was different in terms of diagnosis and degree of treatment-resistance. Indeed, these features can affect the immune profile in terms of both peripheral and central inflammation [46, 47]. Nevertheless, our data suggest that minocycline exerts an antidepressant effect that is already detectable at 4 weeks and that such effect is associated with baseline inflammatory status and possibly with some reduction of inflammation over time, with stronger biological changes that might have been visible with longer treatment.

Of course, the lack of clear changes in immune biomarkers even in the CRP^+^/M group, that shows a significant clinical improvement, may imply the mechanism behind this effect is not related to a reduction of peripheral inflammation (at least not after 4 weeks), and that other pharmacological mechanisms activated by minocycline might be involved. Indeed, as mentioned above, due to its ability to cross the blood-brain barrier, minocycline might act on several inflammatory pathways primarily localised in the CNS and involved in the development of depressive symptoms. In addition to its described effects as anti-oxidant and modulator of several neurotransmitters, minocycline is an inhibitor of microglia activation [10], a possible component of brain neuroinflammatory processes that have been reported in patients with depression [9]. Indeed, a number of preclinical studies have shown the ability of minocycline to ameliorate depressive-like symptoms via suppression of microglia activation [48, 49]. It is therefore possible that minocycline could exert its antidepressant properties through a more direct effect on CNS inflammation, preceding that on peripheral inflammation. So far, a correlation between neuroinflammatory processes and peripheral inflammatory biomarkers has not been found in patients with MDD [9, 50], suggesting possibly the presence of complex and not linear interaction between central and peripheral inflammation, with potentially different timings and dynamics involved in development and regression of central and peripheral inflammatory processes.

Minocycline has also been suggested to inhibit metabolic pathways such as the kynurenine pathway, which is activated during inflammation [13]. Relevant for our study is the well-known activating effect that inflammatory cytokines, in particular IFN-γ, exert on the transcription of IDO, the rate-limiting enzyme of kynurenine pathway of tryptophan metabolism. Indeed, previous data suggest that upregulated production of IFN-γ in the periphery and in the brain can trigger the kynurenine pathway as part of the inflammatory cascade involved in aging and in psychiatric disorders [51]. Therefore, in addition to the well-known effect of minocycline on the inhibition of IDO, our data suggest that minocycline could also inhibit IDO via reduction in the IFN-γ levels, as indicated by the decrease in IFN-γ levels in the minocycline group compared with the placebo group in our study. This is also supported by previous preclinical studies showing that minocycline can reduce the expression of IFN-γ [10].

Our results should be discussed in light of a previous 12-week RCT, by Husain et al., in patients with treatment-resistant depression [18]. In line with this study, we confirmed the efficacy of minocycline in treatment resistant depression, but we added that the basal inflammatory status is also relevant to predict response to minocycline. In the study by Husain and colleagues, the superiority of minocycline over placebo in improving depressive symptoms was found without considering patients’ basal peripheral inflammatory levels. This discrepancy might be due to the fact that the aforementioned study did not find an overall response to placebo and also to the different length of the trial (12 weeks) compared to ours (4 weeks). Interestingly, in the study by Husain and colleagues, treatment differences started to appear at week 4 and became evident by week 8. We hypothesise that patients with lower levels of peripheral inflammation (in our sample those with hsCRP < 3 mg/L) might have a delayed response to minocycline and that a clearer difference between minocycline and placebo could appear with a longer duration of treatment.

The two studies also differ for the severity of baseline depressive symptoms, with patients in the study by Husain et al. showing more severe depressive symptoms than our sample (average baseline HAM-D total score > 30 as opposed to values < 20 in our sample). As the authors explain, placebo response might decline with increasing severity of baseline depression scores [52]. This could also explain why they found minocycline response without taking into account patients’ basal inflammation. Finally, it must be considered that the aforementioned study was conducted in Pakistan while ours had place in London. Thus, the different settings, as well as patients’ heterogeneity might contribute to explain different results.

In line with the same study, we found that minocycline was well-tolerated compared with placebo in terms of side effects, and there was no significant difference in the frequency of adverse events between the two groups.

Finally, our exploratory analyses with secondary outcome clinical measures found no particular difference between minocycline and placebo in the other clinical scales.

Overall, data from our study suggest that minocycline could be a relatively safe and well-tolerated augmentation strategy for MDD, in particular for patients with inflammation-related depression who do not benefit sufficiently from antidepressants alone. Moreover, integrating the measurement of biological markers such as CRP (which is relatively inexpensive) in patients’ first assessments could help identifying potential responders to minocycline.

It is also worth noting that this is the third RCT with positive results on minocycline in unipolar depression. Such evidence suggests that minocycline antidepressant effect might be diagnosis-specific, considering that results in bipolar depression are more conflicting. Indeed, a recent work pointed out that minocycline was not superior to placebo for the acute management of bipolar depression [53]. However, our study also indicates that conventional diagnosis should be complemented with the assessment of biological factors, like the immune markers, in order to identify effective treatments for depression, including anti-inflammatories.

The main strengths of our study were (1) the a priori recruitment of patients with elevated inflammation and (2) the measurement of several inflammatory biomarkers, which had not been performed in previous studies. This enabled us to add knowledge on the relationship between clinical and biological outcomes in immune-related depression treated with minocycline. Moreover, the comparison between complete case (CC) and ITT analysis increased the robustness of the data.

Our results should also be interpreted in light of some limitations, such as the small sample size. Indeed, although our sample size was similar to that of previous RCTs with minocycline, the further division of the sample in 4 groups led to even smaller sizes (ranging from 6 to 12 patients and from 8 to 14 patients per subgroup in the CC and ITT analysis, respectively). Moreover, we could not identify enough patients with treatment response as defined by a 50% reduction in the HAM-D-17 score and we had to consider partial response, instead. This is probably because of the shorter trial duration, i.e., 4 weeks compared with longer RCTs. Another limitation is the lack of follow-up data after the 4 weeks assessment, so that we cannot comment on the long-term efficacy of both minocycline and placebo. Finally, we could not add more clinical information such as the number of failed treatments in patients’ lifetime and in the current episode and the duration of the current episode of depression. This information would have helped to better understand the low response rate in the present study, in terms of 50% reduction in the HAM-D-17 scores.

## Conclusions

In conclusion, we found suggestive evidence that minocycline was a beneficial add-on therapy in a subgroup of MDD patients with levels of hsCRP ≥ 3 mg/L. Such antidepressant effect was independent from changes in peripheral biomarkers and suggests the involvement of other mechanisms, possibly related to central inflammation. Although replications in larger samples are needed, we believe our study has a potentially important clinical impact, as we moved a step towards the identification of personalised treatments for depression.

## Funding and disclosure

This research was funded by the National Institute for Health Research (NIHR) Biomedical Research Centre at South London and Maudsley NHS Foundation Trust and King’s College London. The views expressed are those of the authors and not necessarily those of the NHS, the NIHR, or the Department of Health and Social Care. VM is supported by MQ: Transforming Mental Health (Grant: MQBF1) and by the Medical Research Foundation (grant number MRF-160-0005-ELP-MONDE).

CMP and VM have received research funding from Johnson & Johnson as part of a research programme on depression and inflammation. CMP has received research funding from the Medical Research Council (UK) and the Wellcome Trust for research on depression and inflammation as part of two large consortia that also include Johnson & Johnson, GSK and Lundbeck. AC has in the last 3 years received honoraria for speaking from Lundbeck; honoraria for consulting from Livanova, Lundbeck, Allergan and Janssen; sponsorship for conference attendance from Janssen; and research grant support for work that includes inflammation and depression from the Medical Research Council (UK), Wellcome Trust (UK), the National Institute for Health Research (UK) and Protexin Probiotics International Ltd. The remaining authors have nothing to disclose.

## Supplementary information

CONSORT Flow Diagram for Randomised Clinical Trial

Supplementary Table 1

Supplementary Table 2
