# Supplementary material for: Augmentation therapy with minocycline in treatment-resistant depression patients with low-grade peripheral inflammation: results from a double-blind randomised clinical trial
Source: Neuropsychopharmacology. 2021 Jan 28;46(5):939–48. doi: 10.1038/s41386-020-00948-6 (PMC8096832; doi:10.1038/s41386-020-00948-6)
Supplement: Supplementary file 1 — CONSORT Flow Diagram for Randomised Clinical Trial [file 41386_2020_948_MOESM1_ESM.doc]

**
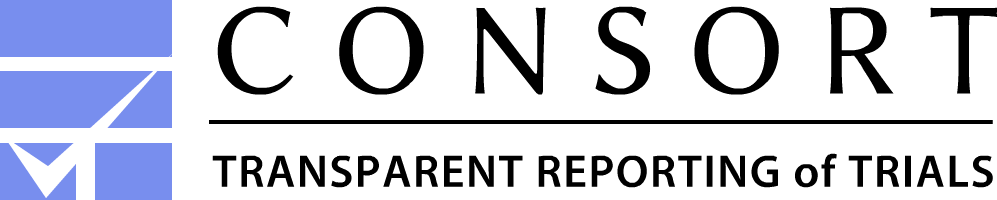
**

**CONSORT 2010 Flow Diagram**

**Allocation**

**Analysis**

**Follow-Up**

**Enrollment**

Assessed for eligibility (n=124)

Excluded (n= 80)

♦  Not meeting inclusion criteria (n=75)

♦  Declined to participate (n=5 )

♦  Other reasons (n= 0)

Analysed (n=18)
♦ Excluded from analysis (n=0)

Lost to follow-up (unknown reasons) (n=2)

Discontinued intervention (side effects) (n=2)

Allocated to minocycline (n=22)

♦ Received allocated intervention (n=22)

♦ Did not receive allocated intervention (give reasons) (n=0)

Lost to follow-up (n=0)

Discontinued intervention (family reasons) (n=1)

Allocated to placebo (n=22)

♦ Received allocated intervention (n=22)

♦ Did not receive allocated intervention (give reasons) (n=0)

Analysed (n=21)
♦ Excluded from analysis (n=0)

Randomized (n= 44)
