# Supplementary material for: Augmentation therapy with minocycline in treatment-resistant depression patients with low-grade peripheral inflammation: results from a double-blind randomised clinical trial
Source: Neuropsychopharmacology. 2021 Jan 28;46(5):939–48. doi: 10.1038/s41386-020-00948-6 (PMC8096832; doi:10.1038/s41386-020-00948-6)
Supplement: Supplementary file 2 — Supplementary Table 1 [file 41386_2020_948_MOESM2_ESM.docx]

**Table S1** Side effects

| ***Side effects*** | ***Minocycline n (%)*** | ***Placebo n (%)*** |
| --- | --- | --- |
| \| Acne \| \| --- \| \| Apathy \| \| Chest palpitation/pain \| \| Constipation \| \| Dizziness \| \| Dyspepsia/indigestion \| \| Flatulence and diarrhoea \| \| Flu-like symptoms \| \| General pain/joint pain \| \| Headache \| \| Insomnia \| \| Light bleeding \| \| Low mood \| \| Extreme happiness \| \| Nausea \| \| No appetite \| \| Skin rash \| \| Sore throat/cold \| \| Tinnitus \| \| Tiredness \| | \| 0 (0) \| \| --- \| \| 0 (0) \| \| 2 (4.5) \| \| 0 (0) \| \| 3 (6.81) \| \| 1 (2.72) \| \| 4 (9.09) \| \| 2 (4.54) \| \| 2 (4.54) \| \| 5 (11.36) \| \| 0 (0) \| \| 0 (0) \| \| 1 (2.72) \| \| 1 (2.72) \| \| 4 (9.09) \| \| 0 \| \| 2 (4.54) \| \| 1 (2.72) \| \| 0 \| \| 1 (2.72) \| | \| 1 (2.72) \| \| --- \| \| 1 (2.72) \| \| 0 (0) \| \| 1 (2.72) \| \| 1 (2.72) \| \| 5 (11.36) \| \| 2 (4.54) \| \| 1 (2.72) \| \| 2 (4.54) \| \| 5 (11.36) \| \| 3 (6.81) \| \| 1 (2.72) \| \| 0 \| \| 0 \| \| 1 (2.72) \| \| 1 (2.72) \| \| 0 \| \| 2 (4.54) \| \| 2 (4.54) \| \| 0 \| |
