# Supplementary material for: Augmentation therapy with minocycline in treatment-resistant depression patients with low-grade peripheral inflammation: results from a double-blind randomised clinical trial
Source: Neuropsychopharmacology. 2021 Jan 28;46(5):939–48. doi: 10.1038/s41386-020-00948-6 (PMC8096832; doi:10.1038/s41386-020-00948-6)
Supplement: Supplementary file 3 — Supplementary Table 2 [file 41386_2020_948_MOESM3_ESM.docx]

|  | **N** | **Mean** | **SD** | **SE** | **MIN** | **MAX** | **Observed vs Imputed Statistics** |
| --- | --- | --- | --- | --- | --- | --- | --- |
| **OBSERVED** | 39 | 13.79 | 5.34 | 0.85 | 2 | 24 | All p>0.05 |
| **IMPUTED*** | 5 | 15.09 | 6.99 | 4.11 | 0 | 32 |  |

**Table S2** Summary statistics for the observed and imputed data for HAM-D-17 at week 4

*summary statistics were calculated using pooled data over 12 imputations

SD=Standard Deviation

SE=Standard Error
